# *Ko*rean *S*troke *Co*hort for functioning and rehabilitation (KOSCO): study rationale and protocol of a multi-centre prospective cohort study

**DOI:** 10.1186/s12883-015-0293-5

**Published:** 2015-03-25

**Authors:** Won Hyuk Chang, Min Kyun Sohn, Jongmin Lee, Deog Young Kim, Sam-Gyu Lee, Yong-Il Shin, Gyung-Jae Oh, Yang-Soo Lee, Min Cheol Joo, Eun Young Han, Yun-Hee Kim

**Affiliations:** Department of Physical and Rehabilitation Medicine, Center for Prevention and Rehabilitation, Heart Vascular and Stroke Institute, Samsung Medical Center, Sungkyunkwan University School of Medicine, 50 Ilwon-dong, Gangnam-gu, Seoul 135-710 Republic of Korea; Department of Rehabilitation Medicine, School of Medicine, Chungnam National University, 282 Munhwa-ro, Jung-gu, Daejeon 301-721 Republic of Korea; Department of Rehabilitation Medicine, Konkuk University School of Medicine, 120-1 Neungdong-ro, Hwayang-dong, Gwangjin-gu, Seoul 143-729 Republic of Korea; Department and Research Institute of Rehabilitation Medicine, Yonsei University College of Medicine, 50-1 Yonsei-ro, Seodaemun-gu, Seoul 120-752 Republic of Korea; Department of Physical and Rehabilitation Medicine, Chonnam National University Medical School, 42 Jebong-ro, Donggu, Gwangju 501-757 Republic of Korea; Department of Rehabilitation Medicine, Pusan National University School of Medicine, Pusan National University Yangsan Hospital, 179 Gudeok-ro, Seo-gu, Busan 602-739 Republic of Korea; Department of Preventive Medicine, Wonkwang University School of Medicine, 895 Muwang-ro, Iksan, Jeonlabuk-do 570-711 Republic of Korea; Department of Rehabilitation Medicine, Kyungpook National University School of Medicine, Kyungpook National University Hospital, 130 Dongdeok-ro, Jung-gu, Daegu 700-721 Republic of Korea; Department of Rehabilitation Medicine, Wonkwang University School of Medicine, 895 Muwang-ro, Iksan, Jeonlabuk-do 570-711 Republic of Korea; Department of Rehabilitation Medicine, Jeju National University Hospital, University of Jeju School of Medicine, 15 Aran 13-gil, Jeju, 690-767 Republic of Korea

**Keywords:** Stroke, Disability, Function, Rehabilitation, Burden

## Abstract

**Background:**

Development of a long-term stroke care plan requires serial assessment of long-term patient function and consideration of caregiver mood. However, to date, few comprehensive cohort studies have included both stroke patients and caregivers.

**Methods/Design:**

KOSCO is a large, multi-centre prospective cohort study for all acute first-ever stroke patients admitted to participating hospitals in nine distinct areas of Korea. This study is designed as a 10-year, longitudinal follow-up investigating the residual disabilities, activity limitations, and quality of life issues arising in patients suffering from first-ever stroke. The main objectives of this study are to identify the factors that influence residual disability and long-term quality of life. The secondary objectives of this study are to determine the risk of mortality and recurrent vascular events in patients with acute first-ever stroke. We will investigate longitudinal health behaviors and patterns of healthcare utilization, including stroke rehabilitation care. We will also investigate the long-term health status, mood, and quality of life in stroke patient caregivers. In addition, we will identify baseline and ongoing characteristics that are associated with our secondary outcomes.

**Discussion:**

KOSCO is a prospective, multi-centre, 10-year longitudinal follow-up study investigating the residual disabilities, activity limitations, and quality of life issues arising in patients suffering from first-ever stroke.

## Background

Although stroke is a world-wide problem [1,2], the burden of stroke is particularly heavy in Korea as the third most common cause of death after heart disease and cancer [3]. Stroke is a medical emergency associated with a very high risk of death in the acute and subacute phases and with a continuous excess risk of death [4]. The greatest predictors of long-term survival after stroke include age, pre-stroke functional level, and functional status at six months after stroke [5,6]. Therefore, it is recommended that stroke patients reduce dependency by six months in order to improve chances of long term survival [5]. While mortality and case fatality rates from stroke have been declining due to risk factor control and improvement in treatment and secondary prevention, the incidence of stroke has increased gradually, in part due to a rapidly aging population [7-9]. Many survivors are left with sequelae to some degree, and disability after stroke remains an important burden to patients, caregivers, and society [10]. It is likely that the burden of post-stroke disability will become an even greater public health problem, considering the expected increase in stroke prevalence [11]. Therefore, studies measuring the burden of stroke will need to include post-stroke disability in addition to mortality.

Many cohort studies of stroke patients have demonstrated the long-term neuropsychological deficits and functional outcomes in United States, Europe, and Asia [10-13]. However, most previous cohort studies have only investigated functional outcomes of stroke during the chronic stage of the disease [10,13]. Stroke recovery is a complex process that probably occurs through a combination of spontaneous and learning-dependent processes, including restitution, substitution, and compensation [14]. This recovery is a dynamic process that cannot be encapsulated at any single timepoint. Also, stroke is a heterogeneous disease by nature. Therefore, long-term prospective studies with long-term assessments in large stroke patient populations are needed in order to rigorously investigate the functional outcomes of this disease.

Stroke survivors usually suffer from impairment in many different domains, including motor, mobility, speech and language, swallowing, vision, sensation, and cognition [11,15]. Although there seems to be a moderate non-linear relation between impairment and function [11,14], the relationship between several different impairments and their combined contribution to function impairment remains poorly understood. Several cohort studies have previously assessed the relationship between these impairment domains and function in stroke patients, albeit without investigating the interaction between functional domains [10-13]. In addition, most studies have not investigated patients in person but rather have relied on telephone interviews. Although a structured telephone interview is widely favored and recommended by researchers [16], it is suboptimal for measuring stroke outcomes. Indeed, it is hard to collect functional data by telephone from stroke patients with moderate to severe levels of language or cognitive impairment [17].

The burden of stroke can extend beyond the stroke patient, and there is a need to better understand its impact on caregivers. Caregivers of stroke survivors tend to have elevated levels of depression during both acute and chronic phases of care [18,19]. Understanding stroke-related patient problems reported by caregivers is important because they are risk factors for caregiver depression, which can be a subsequent risk factor for negative outcomes of the patient [19]. Although caregiver mood may change over time due to learned coping mechanisms, few studies have actually addressed long-term changes in mood and quality of life in caregivers.

Development of a long-term stroke care plan first requires serial assessment of long-term patient function alongside consideration of caregiver mood. We will therefore perform the Korean Stroke Cohort for Functioning and Rehabilitation (KOSCO): a large, multi-centre prospective cohort study on comprehensive assessments of survival rate, recurrence rate, function, mood and quality of life of patients with an ischemic or hemorrhagic stroke, and of their caregivers, by face-to-face interview.

## Methods

### Study design

KOSCO is a large, multi-centre, prospective cohort study for all acute first-ever stroke patients admitted to the participating hospitals in nine distinct areas of Korea. This 10-year longitudinal follow-up study of stroke patients is a prospective multi-centre project that investigates the residual disabilities, activity limitation, and quality of life of patients suffering from first-ever stroke. The study further identifies the factors that influence patient residual disability and long-term quality of life. Written informed consent is obtained from all patients prior to inclusion in the study, and the study protocol is approved by the ethics committees of each hospital.

### Objectives

The main objective of this study is to identify the factors that influence long-term residual disabilities, activity limitations, and quality of life in acute first-ever stroke patients. The secondary objectives are to determine the risks of mortality and recurrent vascular events in patients with acute first-ever stroke. We will investigate long-term health behaviors and healthcare utilization patterns, including stroke rehabilitation care. We will also investigate the changes in health, mood, and quality of life of patient caregivers over time. In addition, we will identify the baseline and follow up characteristics that are associated with our secondary outcomes.

### Study population

All consecutive patients with an acute first-ever stroke, admitted to the representative hospitals in the nine distinct areas of Korea, will be asked to participate in the study. Participating study centres are Samsung Medical Center, Seoul; Severance Hospital, Seoul; Konkuk University Hospital, Seoul; Chungnam National University Hospital, Daejeon; Chonnam National University Hospital, Gwangu; Pusan National University Yangsan Hospital, Yangsan; Kyungpook National University Hospital, Deagu; Wonkwang University Hospital, Iksan; and Jeju National University Hospital, Jeju.

The followings are criteria for inclusion in the study:

1. First-ever acute stroke (ischemic stroke or intracerebral hemorrhage) with corresponding lesion and/or evidence of acute arterial occlusion on CT (A)- or MRI/A-scan.
2. Age *≥* 19 years at onset of stroke.
3. Onset of symptoms within seven days prior to inclusion.

Acute stroke is defined as a rapidly evolving, focal neurological deficit persisting for more than 24 hours. On the basis of neuroimaging, stroke is further classified as ischemic or hemorrhagic stroke. Hemorrhagic transformation of an ischemic stroke will be classified as an ischemic stroke.

The followings are criteria for exclusion in the study:

1. Transient ischemic attack.
2. History of stroke.
3. Traumatic intracerebral hemorrhage.
4. Not Korean.

Transient ischemic attack is defined as a rapidly evolving, focal neurological deficit with only vascular etiology lasting less than 24 hours.

### Procedures

All eligible patients will be recruited at the time of stroke evaluation. Patient recruitment is planned over a three to four year period, and we intend to include 7,500 patients in the cohort. After providing written informed consent, patients will formally enter the study. If the patient is unable to provide informed consent, consent will be obtained from the patient’s legally authorized representative.

**Figure 1:**
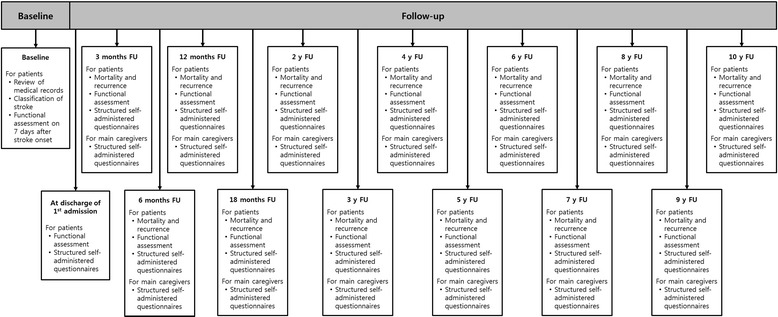
**Flowchart of the KOSCO study.**

After baseline assessment, patients will be assessed during face-to-face interviews at 7 days, discharge, 3 months, 6 months, 12 months, 18 months and 24 months after stroke onset and every year thereafter for the duration of the study period. Figure 1 depicts a flowchart of the study design.

#### Baseline review of medical records

At baseline, a complete enumeration survey of all patients will be performed using a review of medical records at first admission. Survey items include demographic data such as age, sex, education, marital status, and employment. Presence of cerebrovascular risk factors will be assessed by standardized, structured questionnaires and will be classified according to the current guidelines of the American Heart Association [20]. Comorbidity will be assessed using the Charlson comorbidity index [21]. Initial stroke severity will be recorded at the time of hospital arrival using the National Institute of Health Stroke Scale (NIHSS) [22] and Glasgow Coma Scale [23] for ischemic and hemorrhagic strokes, respectively. Functional performance prior to the index event will be assessed by modified Rankin scale (mRS) [24].

Clinical signs, symptoms, and duration of stroke will be recorded using review of medical records. Physical examination findings and laboratory measures will also be recorded. Furthermore, the course of the disease during admission will be reported, including information on medication use, (intra-venous or arterial) thrombolysis, or other treatments and complications. Development of recurrent cerebrovascular event or neurologic aggravation will also be reported. In addition, details of the first admission course of all patients, including consultation to rehabilitation department, inpatient rehabilitation treatment, transfer to rehabilitation department, discharge destination, and discharge status, will be recorded. Table 1 shows an overview of all planned investigations from medical record review.

**Table 1:** **Baseline medical record assessments of stroke patients**

	**Assessment**
Demographics	Age
	Sex
	Dominant hand
	Marital status
	Years of education
Medical history	Cerebrovascular risk factors
	Pre-stroke functional level
	Comorbidities
Stroke characteristics	Etiology
	Territory
	Symptom duration
	Initial National Institute of Health Stroke Scale in ischemic stroke
	Initial Glasgow Coma Scale in hemorrhagic stroke
Physical examination	Height
	Weight
	Body mass index
	Waist circumference
	Initial body temperature
	Initial blood pressure
	Initial heart rate
Laboratory measures	Hyperglycemia
	C-reactive protein
	Erythrocyte sedimentation rate
	Fibrinogen
	Leukocyte count
	Creatinine
	Homocystein
	Hemoglobin
	Hematocrit
	Platelet
	Aspartate aminotransferase (AST)/Alanine aminotransferase (ALT)
	Protein/Albumin
	Cholesterol
	Triglyceride
	High density lipoprotein (HDL)
	Low density lipoprotein (LDL)
Stroke care	Initial admission site
	Stroke treatments
	Consult to rehabilitation department
	Transfer to rehabilitation department
	Rehabilitation treatments
Discharge	Discharge destination
	Discharge status
	Progress of stroke during first admission
	Complications during first admission

#### Classification of stroke: etiology and neuro-imaging

Etiology of ischemic stroke will be classified according to the TOAST criteria [25]. Etiology of intracerebral hemorrhage will be classified according to previous stroke classification [25]. Etiology will be based on neuro-imaging, medical history, and use of medication. All patients will undergo neuroimaging, and CT-angiography, MR-angiography, or ultrasound will be performed according to standard clinical practice. CT- and MRI-scans will be reviewed by neuroimaging specialists in each institute. Ischemic strokes will be classified according to arterial territory and as lacunar or territorial. Intracerebral hemorrhage will be classified as infratentorial (brainstem or cerebellar) or supratentorial hemorrhage (lobar, deep or ventricular), with or without ventricular involvement. Lobar hemorrhage will be further subclassified as frontal, temporal, parietal, or occipital.

#### Follow-up

All patients will be approached for follow-up assessment using a two-step approach. First, all patients will be contacted by telephone. In cases of an invalid phone number, a second telephone call will be made to contact caregivers of patients. Information on patient vital status will be obtained in this way through telephone interview of patient or caregivers. Subsequently, all living patients will be invited to visit our research centre for functional assessment batteries, structured self-administered questionnaires, and face-to-face interviews (Table 2). If patients are not able to visit our research centre, these same investigations will be performed at their homes. Main caregivers will also visit our research centre or be visited by our researchers for structured self-administered questionnaires and face-to-face interviews (Table 3).

**Table 2:** **Neuropsychological assessments and structured questionnaires for stroke patients of the KOSCO study**

**Domain**	**Assessment**
Stroke severity	National Institute of Health Stroke Scale
Activities of daily living	Korean modified Barthel Index
	Functional Independence Measure
Cognition function	Korean Mini-Mental State Examination
Motor function	Fugl-Meyer Assessment
	modified Ashworth scale
	9-hole peg board test
Mobility function	Functional Ambulatory Category
Swallowing function	American Speech-Language-Hearing Association National Outcome Measurement System Swallowing Scale
Language function	Korean Version of Frenchay Aphasia Screening Test
Disability	modified Rankin scale
Mood	Geriatric depression scale-short form
Quality of life	Euro Quality of Life-5D
Subjective health condition	Psychosocial Well-being index-short form
	Reintegration to Normal Living Index
Demographics	Occupation
	Marital status
	Living conditions
Lifestyle habits	Alcohol consumption
	Smoking
	Exercise
Health care utilization	General medical services
	Rehabilitation services

**Table 3:** **Structured questionnaires for main caregivers of the KOSCO study**

	**Assessment**
Demographics	Age
	Sex
	Dominant hand
	Marital status
	Year of education
	Living conditions
	Occupation
Knowledge of stroke	Knowledge of stroke questionnaires
Mood	Geriatric depression scale-short form
Quality of life	Euro Quality of Life-5D
Subjective health condition	Psychosocial Well-being index-short form
Subjective caregiving burden	Subjective caregiving burden questionnaires

Patient follow-up has begun in 2012 and is planned to finish by the end of 2021.

### Functional assessment battery

#### Baseline

Seven days after onset of stroke, baseline evaluation will be performed using face-to-face functional assessment, including K-NIHSS for stroke severity, Korean Mini-Mental State Examination (K-MMSE) [26] for cognitive function, Fugl-Meyer Assessment (FMA) [27] for motor function, Functional Ambulatory Category (FAC) [28] for mobility and gait, the American Speech-Language-Hearing Association National Outcome Measurement System Swallowing Scale (ASHA-NOMS) [29] for swallowing function, the Korean Version of the Frenchay Aphasia Screening Test (K-FAST) [30] for language function, and mRS for general functional level.

#### During first admission

At discharge, all patients will be evaluated using a face-to-face functional assessment battery that includes the Functional Independence Measure [31], Korean modified Barthel Index [32], K-NIHSS, K-MMSE, FMA, FAC, ASHA-NOMS, K-FAST, and mRS.

For patients to be transferred to the rehabilitation department, this same discharge evaluation will also be performed on the day of their transfer.

#### Follow-up

At every follow-up visit, all patients will undergo the same evaluation that was performed at discharge using face-to-face functional assessment battery.

### Structured self-administered questionnaires for patients

At discharge, all patients will complete structured self-administered questionnaires and undergo a face-to-face interview for mood and quality of life evaluation with the Geriatric depression scale-short form (GDS-SF) [33] and Euro Quality of Life (EQ)-5D [34].

For patients to be transferred to the rehabilitation department, same discharge questionnaires and interview will also be performed on the day of their transfer.

At every follow-up visit, all patients will complete structured self-administered questionnaires and undergo a face-to-face interview. Standardized questionnaires on patient demographics, marital status, occupation, living conditions, stroke recurrence, and lifestyle habits (i.e., alcohol consumption, smoking, exercise) will be administered. Alcohol consumption will be defined as units per day, and the frequency of alcohol consumption will be noted. Cigarette smoking behavior will be defined as never, former, or current. Subsequently, former and current smoking behavior will be quantified as the number of pack-years, calculated as the number of packs of cigarettes smoked per day multiplied by the number of years a participant has smoked. Exercise will be expressed in amount and frequency of physical activity per week. Health care utilization information will also be collected via standardized questionnaires. Patients will report if they have had an inpatient rehabilitation facility stay, a skilled nursing facility stay, used home health services, used outpatient rehabilitation services, the number of physician office visits, and/or were readmitted to a hospital after discharge from the initial stroke-induced hospitalization. For those patients that have difficulty in completing the survey, the main caregiver will complete it in their stead. In addition, at each follow-up visit, patients will complete structured self-administered questionnaires and undergo face-to-face interviews to assess for mood, quality of life, and subjective health conditions including GDS-SF, EQ-5D, Psychosocial Well-being index-short form (PWI-SF) [35] and Reintegration to Normal Living Index (RNLI) [36] assessments.

### Structured self-administered questionnaires for main caregivers

At every follow-up, all caregivers will complete structured self-administered questionnaires and undergo a face-to-face interview. Standardized questionnaires on demographics, marital status, occupation, living conditions, and life style habits will be administered. Also, standardized questionnaires will include questions on the subjective caregiving burden and knowledge of stroke. In addition, all caregivers will complete structured self-administered questionnaires and undergo a face-to-face interview for mood, quality of life, subjective health condition including GDS-SF, EQ-5D, and PWI-SF.

### Statistical analysis

Multivariate analysis by linear or logistic regression will be performed to identify independent predictors of the primary outcome. A repeated measures ANOVA will be used to investigate change in continuous variables over time points. In case of skewed distributions which cannot be normalized, corresponding nonparametric tests will be used. Cumulative risk of mortality and recurrent vascular events will be estimated with Kaplan-Meier analysis. Cox proportional hazard models will be used to calculate the mortality and recurrent vascular events in the follow-up period, with adjustments for the necessary covariates. The relative risk (hazards ratios) will be calculated with their corresponding 95% confidence intervals.

## Discussion

KOSCO is a prospective, multi-centre, ten-year follow-up study of the residual disabilities, activity limitations, and quality of life of first-ever stroke patients. In KOSCO study, 7,500 first-ever stroke patients will be consecutively recruited from the participating hospitals in the nine distinct areas of Korea over three years. The main goal of this cohort study is to identify the factors that influence patient residual disability and long-term quality of life. In addition, we intend to determine the factors that influence the long-term mood, and quality of life in stroke patient caregivers.

This study has much strength, including its multi-centre prospective design with long-term multiple follow-up assessments. The multi-centre design allows for a large sample size, and therefore greater statistical power, drawing from a population that covers much of Korea. The prospective design allows us to obtain an accurate evaluation of the residual disabilities, activity limitations, and quality of life of first-ever stroke patients. Another advantage of our study is that it will include patients with hemorrhagic and ischemic stroke. These inclusion criteria allow us to study the entire spectrum of stroke. Additionally, since patients will be eligible for inclusion in this study only if they have positive brain imaging corresponding with neurological deficits, the KOSCO study will have a clearly diagnosed population without potential for misclassification of patients.

Most importantly, the KOSCO study has three key strengths that distinguish it from previous large multi-centre cohort studies. First, unlike previous cohort studies, patient evaluation in this study will be performed by the face-to-face assessment rather than telephone interview. This approach will allow us to more accurately evaluate patients. Second, surveys will be given to both caregivers and patients. This will allow us to gain insight on the greater burden of stroke. Finally, lifestyle and healthcare utilization information, including on stroke rehabilitation, will also be collected prospectively. This can provide insight on the optimal time points for interventions and could ultimately lead to improved guidelines for chronic stroke patients.

Completion of this study will contribute to better understanding of the residual disabilities, activity limitations, and quality of life issues that arise in acute first-ever stroke patients. It may also provide physicians with better information on how to better treat stroke patients. Given the growing burden of stroke, identification of the factors that influence residual disability and long-term quality of life will be necessary for improving healthcare outcomes for stroke patients. In addition, it may contribute to better understanding of the mood, and quality of life of patient caregivers. Finally, the results of this cohort study can be used to inform healthcare policy decisions that could reduce the burden of post-stroke morbidity.

## Abbreviations

KOSCO: Korean Stroke Cohort for Functioning and Rehabilitation
K-NIHSS: Korean version of the National Institute of Health Stroke Scale
mRS: Modified Rankin scale
K-MMSE: Korean Mini-Mental State Examination
FMA: Fugl-Meyer assessment
FAC: Functional ambulatory category
ASHA-NOMS: American Speech-Language-Hearing Association National Outcome Measurement System Swallowing Scale
K-FAST: Korean Version of the Frenchay Aphasia Screening Test
GDS-SF: Geriatric depression scale-short form
EQ: Euro quality of life
PWI-SF: Psychosocial Well-being index-short form
RNLI: Reintegration to Normal Living Index
